# Supplementary material for: A Critical View of the Application of the APEX Software (Aqueous Photochemistry of Environmentally-Occurring Xenobiotics) to Predict Photoreaction Kinetics in Surface Freshwaters
Source: Molecules. 2019 Dec 18;25(1):9. doi: 10.3390/molecules25010009 (PMC7017383; doi:10.3390/molecules25010009)
Supplement: Supplementary file 1 [file molecules-25-00009-s001.zip › 3.2.4_gcc-4.4.0.zip!/3.2.4_gcc-4.4.0/3.2.4_gcc-4.4.0/doc/octave/html/interpreter/Hashing-Functions.html]

Hashing Functions - Untitled


Previous: System Information,
Up: System Utilities


---

### 34.12 Hashing Functions

It is often necessary to find if two strings or files are
identical. This might be done by comparing them character by character
and looking for differences. However, this can be slow, and so comparing
a hash of the string or file can be a rapid way of finding if the files
differ.

Another use of the hashing function is to check for file integrity. The
user can check the hash of the file against a known value and find if
the file they have is the same as the one that the original hash was
produced with.

Octave supplies the `md5sum` function to perform MD5 hashes on
strings and files. An example of the use of `md5sum` function might
be

```
     if exist (file, "file")
       hash = md5sum (file);
     else
       # Treat the variable "file" as a string
       hash = md5sum (file, true);
     endif
```

— Loadable Function: **md5sum** (file)  
— Loadable Function: **md5sum** (str, opt)  
> Calculates the MD5 sum of the file file. If the second parameter
> opt exists and is true, then calculate the MD5 sum of the
> string str.
